# Experimental Study on Calibration of Amplitude-Frequency Measurement Deviation for Microseismic Sensors in Coal Mines

**DOI:** 10.3390/s23208420

**Published:** 2023-10-12

**Authors:** Zepeng Han, Linming Dou, Zonglong Mu, Jinrong Cao, Yanjiang Chai, Shuai Chen

**Affiliations:** 1 State Key Laboratory of Coal Exploration and Intelligent Mining, China University of Mining and Technology, Xuzhou 221116, China; hazp@cumt.edu.cn (Z.H.);; 2 School of Mines, China University of Mining and Technology, Xuzhou 221116, China; 3 School of Mechanical and Mining Engineering, University of Queensland, St. Lucia, QLD 4072, Australia

**Keywords:** microseismic monitoring system, microseismic sensor, amplitude–frequency calibration, positioning accuracy, vibration table

## Abstract

Microseismic monitoring systems (MMS) have become increasingly crucial in detecting tremors in coal mining. Microseismic sensors (MS), integral components of MMS, profoundly influence positioning accuracy and energy calculations. Hence, calibrating these sensors holds immense importance. To bridge the research gap in MS calibration, this study conducted a systematic investigation. The main conclusions are as follows: based on calibration tests on 102 old MS using the CS18VLF vibration table, it became evident that certain long-used MS in coal mines exhibited significant deviations in frequency and amplitude measurements, indicating sensor failure. Three important calibration indexes, frequency deviation, amplitude deviation, and amplitude linearity are proposed to assess the performance of MS. By comparing the index of old and new MS, critical threshold values were established to evaluate sensor effectiveness. A well-functioning MS exhibits an absolute frequency deviation below 5%, an absolute amplitude deviation within 55%, and amplitude linearity surpassing 0.95. In normal operations, the frequency deviation of MS is significantly smaller than the amplitude deviation. Simplified waveform analysis has unveiled a linear connection between amplitude deviation and localization results. An analysis of the Gutenberg–Richter microseismic energy calculation formula found that the microseismic energy calculation is influenced by both the localization result and amplitude deviation, making it challenging to pinpoint the exact impact of amplitude deviation on microseismic energy. Reliable MS, as well as a robust MS, serve as the fundamental cornerstone for acquiring dependable microseismic data and are essential prerequisites for subsequent microseismic data mining. The insights and findings presented here provide valuable guidance for future MS calibration endeavors and ultimately can guarantee the dependability of microseismic data.

## 1. Introduction

Microseismic events, arising as concomitant phenomena of rock mass deformation, crack initiation, and crack propagation, are earthquakes characterized by a magnitude below 3.0. These events possess less energy and a lower signal-to-noise ratio (SNR) in comparison to larger, natural earthquakes. The Microseismic Monitoring System (MMS) serves as a comprehensive record documenting the temporal and spatial attributes of microseismic events [1]. Since its inception in the 1970s and its commercialization circa 2000, MMS has proved invaluable for understanding underground processes [2]. While its most common and notable use has been hydraulic-fracture mapping, it is also used for monitoring mining-induced microseismic, gas outbursts, and water inrush [3]. China is the world’s largest coal producer, and 85% of its output comes from underground coal mines [4]. In China, a growing number of deep coal mines have been under construction in recent years due to the shallow depletion of coal resources. Deep underground coal mines are more evidently featured by “high static load, strong dynamic load” [5]. It leads to many problems associated with Mine pressure problems at great depth, with coalburst as the most prominent one. Coalburst not only undermines underground roadways and damages equipment but also seriously threatens personnel safety [6]. It has become a major technical bottleneck in China’s future deep underground coal mine engineering [7]. Currently, MMS is the most effective and promising means to predict coalburst [8,9]. According to statistics, 144 coal burst-prone mines in China have installed various types of MMS [10]. Microseismic sensor (MS) is the core element of MMS, which converts mechanical vibration into electrical signals using the electromagnetic induction principle. The quality of MS directly affects the accuracy of source location and energy calculation of MMS. Usually, each set of MMS needs to be equipped with dozens of MS. Whether the MS can maintain accurate measurement after long-term use in the underground is the key to the regular use of MMS. 

The history of microseismic monitoring dates back to 1938 when the U.S. Bureau of Mines researched to establish a correlation between seismic wave velocity and pillar load [11]. During this research, a significant uptick in the rate of seismic events was notably observed in the period leading up to structural failure. In the early 1960s, researchers from South Africa and the United States began to study and utilize MMS to monitor rockburst locations. Since the mid-1980s, over 20 rockburst-prone mines in Canada have been installed with MMS so that severe rockburst disasters can be routinely monitored [12]. Luo et al. applied this technique to undertake microseismic monitoring at two long-wall mines [13]. Rutledge et al. successfully conducted a hydraulic fracture operation in the Cotton Valley gas field in East Texas, USA, in the same year [14]. With the development of technology relating to electronics, data storage, data remote transmission, and data processing, the microseismic monitoring system was improved from an analog signal type to a full digital type in the 1990s [15]. In China, the earliest relevant research of MMS can be traced back to the 581 microseismic meter developed by the Institute of Geophysics of the Chinese Academy of Sciences in 1959 [16]. Its sensor was modified from the earthquake domain, which had certain limitations on mining-induced microseismic monitoring. In 1995, Huafeng Coal Mine cooperated with the Chinese Geophysical Society to design an MMS, continuously monitored for over ten years. Starting in 2004, with the improvement of the domestic coal market, more than 20 sets of MMS have been established in various mining enterprises [17,18,19,20]. After 2004, coal enterprises began introducing SOS MMS and ARAMISM/E MMS from Poland, which promoted improved mining monitoring technology and equipment in China [21]. According to incomplete statistics, the domestic coal mine has currently been equipped with about 100 sets of SOS MMS, about 60 sets of ARAMISM/E MMS, and more than 40 sets of other models of MMS. 

During its initial phase, China predominantly concentrated on producing complete MMS equipment. Limited attention was given to fundamental MS, with most MMS relying on established accelerometers or velocity sensors from the earthquake domain. This directly impacted the resolution, sensitivity, and reliability of domestically developed MMS systems in coal mines [22]. According to the market share of each type of MMS in coal mines, it is inferred that the most used MS is the DLM-type of SOS, followed by the G series of ARAMISM/E, and the remaining small part is the other sensor. DLM-type and G series are vertical single-component speed sensors. DLM-type requires installation on the roadway floor, G series is divided into three types: G_Vu_, G_Vd_, and G_H_, installed in the roof, floor, and side of the roadway, respectively. Vertical single-component velocity MS is mainly used in coal mines, and three-component sensors are applied less. So, the MS mentioned in this paper refers to vertical single-component velocity MS. 

Because of the fundamental status of the MS and its importance in the MMS, the Chinese national standard puts forward the calibration requirements for the MS, requiring the calibration of the MS every two years to ensure the reliability of the MS [23]. A comprehensive review of the existing literature reveals a scarcity of research dedicated to MS calibration. This paper presents the principles of MS calibration, introduces three innovative calibration indices, and conducts calibration testing on the DLM-type MS using the CS18VLF vibration table. Moreover, the article establishes a critical value for evaluating sensor qualification using comparative tests. Furthermore, the study delves into the effects of frequency and amplitude deviation on microseismic location accuracy and energy calculations. By employing this approach, a fundamental benchmark is established to evaluate the efficacy of DLM-type MS. These preliminary findings serve as a valuable reference point for calibrating MS in coal burst-prone mines and relevant scientific research institutions. 

## 2. Experimental Method

### 2.1. Introduction of Microseismic Sensor

**Figure 1:**
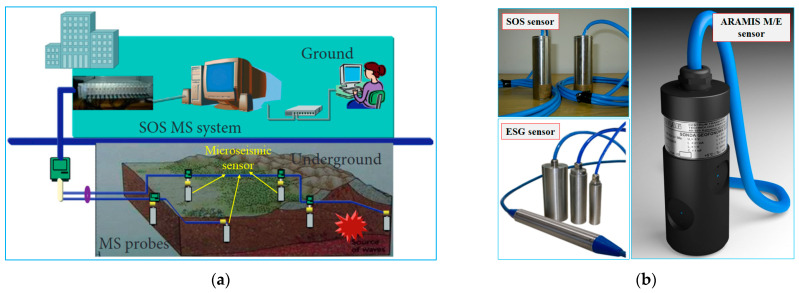
Microseismic monitoring system and microseismic sensor: (**a**) Configuration of SOS microseismic monitoring system within a mine; (**b**) microseismic sensors designed for coal mines.

MMS is the placement of MS in advantageous positions from which small earthquakes (microseisms) induced by some downhole process can be detected and located to provide geometric and behavioral information about the process [24]. MMS was widely used to forecast dynamic disasters (such as rock bursts, coal bursts, and gas outbursts). The working process of MMS can be divided into five parts: vibration generation, vibration propagation, signal acquisition, signal transmission, and signal analysis (Figure 1a). In China, SOS MMS (designed by Central Mining Institute in Poland) and ARAMISM/E MMS (designed by EMAG in Poland) are widely used in microseismic monitoring in coal mines, ESG MMS (designed by Engineering Seismic Group in Canada) is also equipped in a few coal mines. Figure 1b shows MMS’s vertical component sensors.

**Figure 2:**
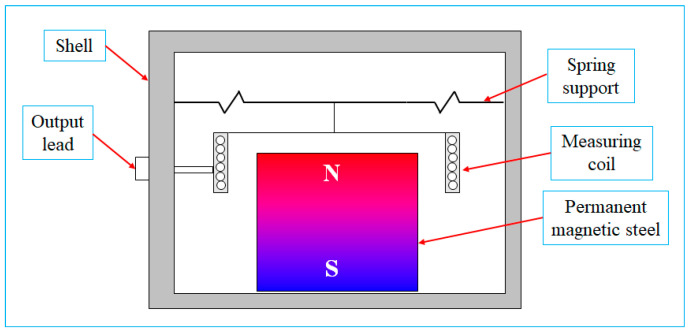
Principles of magnetoelectric microseismic sensors.

Figure 2 illustrates the operational mechanism of the MS relying on electromagnetic induction. The pivotal component of the sensor is a permanent magnet, which generates a stable magnetic field. The measuring coil is linked to one extremity of a spring support, with the opposing end affixed to the shell. The MS is affixed to the roadway wall. As the propagated vibrations reach the MS, the permanent magnet and the shell undergo synchronized vertical oscillations that replicate the motion of the measured roadway. Supported by the spring support, the measuring coil remains relatively immobile due to inertial forces. This stationary state enables the measuring coil to intersect the magnetic field, thereby inducing an electromotive force. Within the operational range of the MS, the electromotive force maintains a direct proportionality to the vibration velocity of the roadway. The electrical signal is conveyed to the data acquisition device, where the energy exchange formula facilitates the computation of vibration parameters.

### 2.2. Preparation of Old Microseismic Sensor

**Figure 3:**
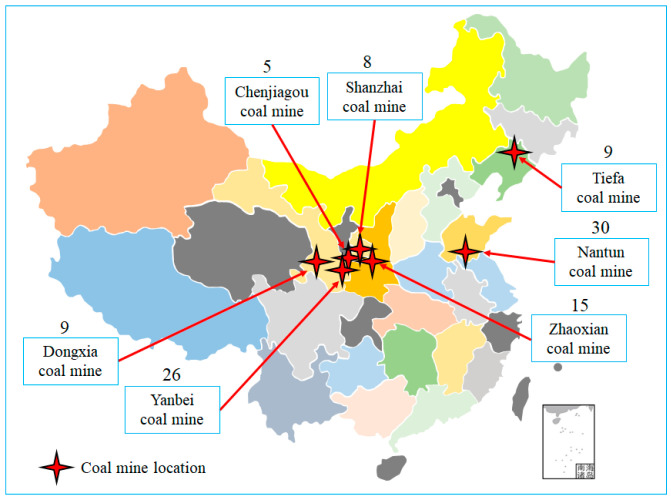
Geographical distribution of sampled coal mines and sample count.

The MS utilized in the test were DLM-type MS sourced from seven coal burst-prone mines after undergoing underground usage for a minimum duration of one year. Each sensor underwent an initial screening process to verify its circuitry’s integrity and compatibility for attachment to the screws. Figure 3 displays the geographical distribution of the sampled coal mines and the respective MS number within each mine. A total of 102 SOS sensors were available for testing purposes. 

### 2.3. Calibration Instrumentation and Scheme Design

#### 2.3.1. Calibration Principles and Equipment

MS calibration can be categorized into absolute and comparative methods [25]. The absolute method measures the fundamental unit of movement, such as time or length. An absolute measurement necessitates the use of a laser interferometer, which can incur substantial costs. The comparative method gauges the motion of a vibration system by comparing it with a standard sensor, commonly referred to as a reference sensor. Standard sensors are usually subjected to absolute calibration using laser interferometry or possess traceability and documented uncertainty, often select accelerometers. 

**Figure 4:**
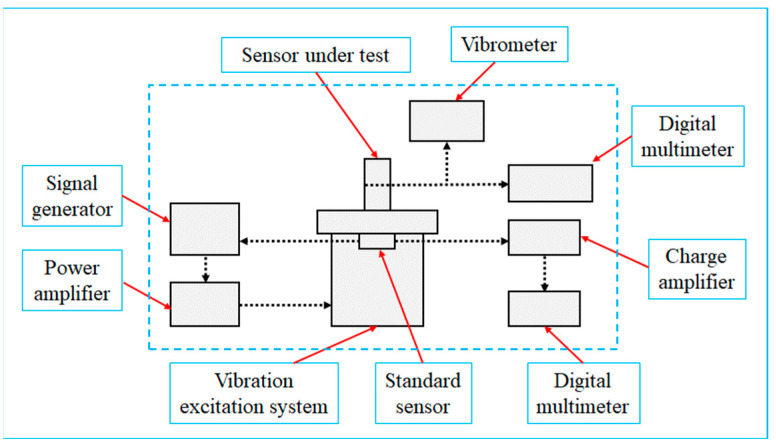
Principle of calibration using the comparative method.

The system connection through the comparative method is illustrated in Figure 4. The signal generator produces electrical signals of a specific frequency and vibration velocity, which are subsequently amplified by the power amplifier before being fed into the vibration excitation system. The standard sensor detects the vibration excitation system’s vibration state, provides feedback to the signal generator, and employs a negative feedback mechanism to regulate the vibration signal. The standard sensor enhances the electric signal using a charge amplifier and transmits it to a digital multimeter. The standard sensor’s vibration velocity and frequency can be exhibited after signal conversion and data processing. The sensor under test is mounted onto the vibration excitation system to gather the vibration parameters. Upon establishing a connection with the sensor, the digital multimeter showcases the vibration frequency, velocity, and other parameters measured by the sensor. Ultimately, a comparison is made between the vibration parameters of the signal generator and the sensor under test to verify the operational status of the MS.

**Figure 5:**
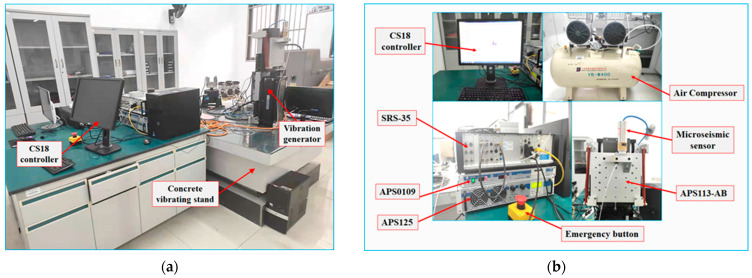
Test environment and vibration excitation system: (**a**) Test environment; (**b**) CS18VLF vibration table.

MS are considered precision instruments, necessitating a meticulously controlled testing environment. According to the Chinese industry standard document, the construction of the concrete vibration table base must adhere to stipulated guidelines [26]. Furthermore, ensuring that the testing environment and its surroundings are devoid of vibrations, impacts, strong electric fields, strong magnetic fields, and high-intensity sound fields is imperative. This assurance establishes an environment for testing the MS that is maximally free from interference. The testing environment for the MS is depicted in Figure 5a. 

The experiment utilizes the CS18VLF low-frequency vibration system from SPEKTRA, a German company. Figure 5b depicts the equipment’s primary components. The performance of the CS18VLF relies on APS113-AB, a force-balanced long-stroke vibration exciter. This exciter fulfills the criteria outlined in the Chinese national standard [27]. It can be employed to inspect and assess accelerometers and other motion sensors. APS113-AB is characterized by a lightweight aluminum housing and a grounded steel table with a matte finish. A comprehensive display of APS113-AB’s performance is presented in Table 1.

#### 2.3.2. Design of Calibration Scheme

**Figure 6:**
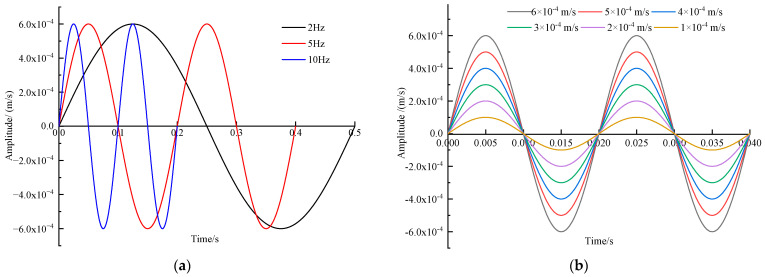
Input waveform for amplitude–frequency calibration: (**a**) Input waveform for frequency calibration; (**b**) input waveform for amplitude calibration.

Precisely capturing the frequency and amplitude of vibration signals stands as the core function of an MS, directly influencing the reliability of an MMS. Within the operational frequency range of the DLM-type MS (0.1~200 Hz), we chose seven representative frequencies for calibration: 2, 5, 10, 20, 50, 100, and 150 Hz. The selected amplitude during the frequency calibration was 6 × 10^−4^ m/s, falling within the functional amplitude range of the DLM-type MS (1 × 10^−6^ m/s ~ 6.4 × 10^−4^ m/s). For the amplitude calibration, we held the frequency steady at 50 Hz and utilized the CS18VLF to generate six distinctive amplitudes: 6 × 10^−4^, 5 × 10^−4^, 4 × 10^−4^, 3 × 10^−4^, 2 × 10^−4^, and 1 × 10^−4^ m/s, respectively. Standard sinusoidal waveforms were chosen for both frequency and amplitude calibration; Figure 6a,b depict the input waveforms utilized for frequency and amplitude calibration. 

The central steps of the calibration test comprise the following: (1) Configuring the frequency and amplitude parameters within the CS18 controller to produce specific frequency and amplitude vibrations on the APS113-AB; (2) acquiring and documenting the output value generated by the MS; (3) processing the data collected by the MS in line with the index calculation formula and subsequently comparing it with the input data.

## 3. Analysis of Experimental Results

A total of 102 MS from diverse coal mines were tested under uniform environmental conditions and equipment. The waveforms captured by the MS units were subjected to Fast Fourier Transform (FFT) processing to extract the vibration frequency. The electrical signal was transformed into velocity using the transduction formula, ultimately yielding the maximum vibration velocity (amplitude).

### 3.1. Amplitude–Frequency Calibration Index

The frequency deviation index was formulated to assess the efficacy of MS during frequency calibration. This index quantifies the disparity between the measured frequency of the MS and the set frequency of the CS18VLF, thereby determining the frequency deviation. The calculation method can be described as follows:

(1) $$\delta_{f}=\frac{f_{i}-f_{r}}{f_{r}}\times 100\%$$

where $\delta_{f}$ is frequency deviation (%), $f_{i}$ is the measured frequency of the tested MS (Hz), and $f_{r}$ is the standard frequency of the CS18VLF system (Hz).

To evaluate the performance of MS in amplitude detection, two parameters have been formulated: amplitude deviation and amplitude linearity. The amplitude deviation calculation follows a similar approach to frequency deviation, making it applicable for assessing the relative accuracy of MS measurements in terms of amplitude. Drawing inspiration from the calculation method of the correlation coefficient, amplitude linearity enables the assessment of whether an MS can maintain consistent errors when capturing vibrations of varying intensities. The calculation method for amplitude deviation is defined using Equation (2), whereas the calculation method for amplitude linearity is delineated using Equation (3):

(2) $$\delta_{a}=\frac{x_{i}-x_{r}}{x_{r}}\times 100\%$$

where $\delta_{a}$ is amplitude deviation (%), $x_{i}$ is the measured amplitude of the tested MS (m/s), and $x_{r}$ is the standard amplitude of the CS18VLF (m/s).

(3) $$R_{a}=\frac{n\sum x_{ii}x_{ri}-\sum x_{ii}\sum x_{ri}}{\sqrt{n\sum x_{ii}^{2}-{\left(\sum x_{ii}\right)}^{2}}\sqrt{n\sum x_{ri}^{2}-{\left(\sum x_{ri}\right)}^{2}}}$$

where $R_{a}$ is amplitude linearity, $x_{ii}$ is the measured amplitude of the tested MS (m/s), $x_{ri}$ is the standard amplitude of the CS18VLF (m/s), and n is equal 6. 

### 3.2. Frequency Calibration Result Analysis

**Figure 7:**
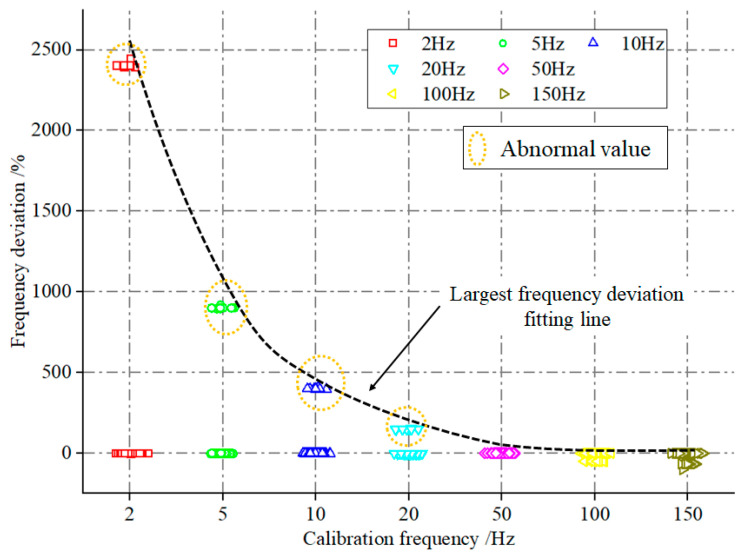
Statistical distribution of tested sensor frequency deviation.

Frequency calibration tests were conducted on 102 existing MS utilizing the test scheme outlined in Section 2.3.2. Subsequently, the frequency deviation index was computed using Equation (1). The aggregated statistical outcomes are presented in Figure 7. As depicted in Figure 7, the frequency deviation among most MS is small, with fluctuations around 0%. Nonetheless, a few MS exhibit frequency deviations beyond the accepted range. For instance, during the 2 Hz calibration, the largest frequency deviation for 11 MS approaches 2500%. Interestingly, the maximum frequency deviation demonstrates a descending pattern with escalating calibration frequencies. This phenomenon could be attributed to the rise in the denominator of Equation (2) resulting from the elevation of the calibration frequency. 

**Figure 8:**
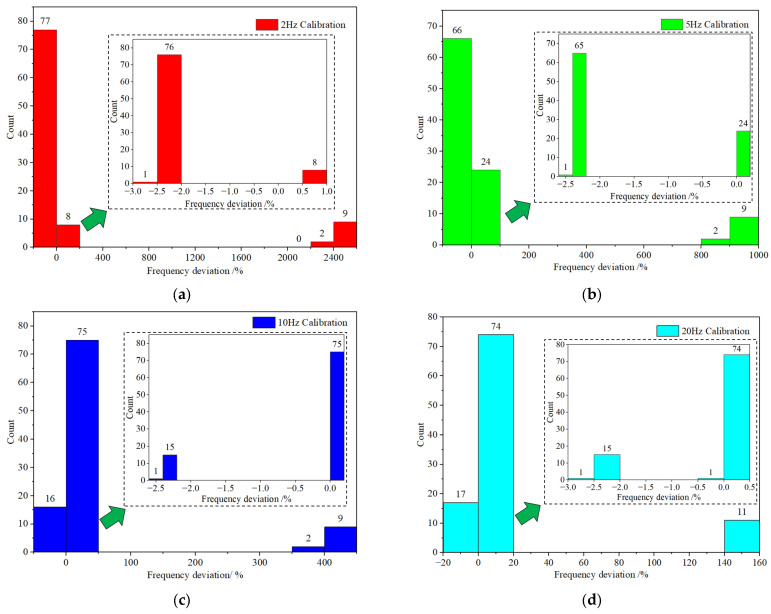

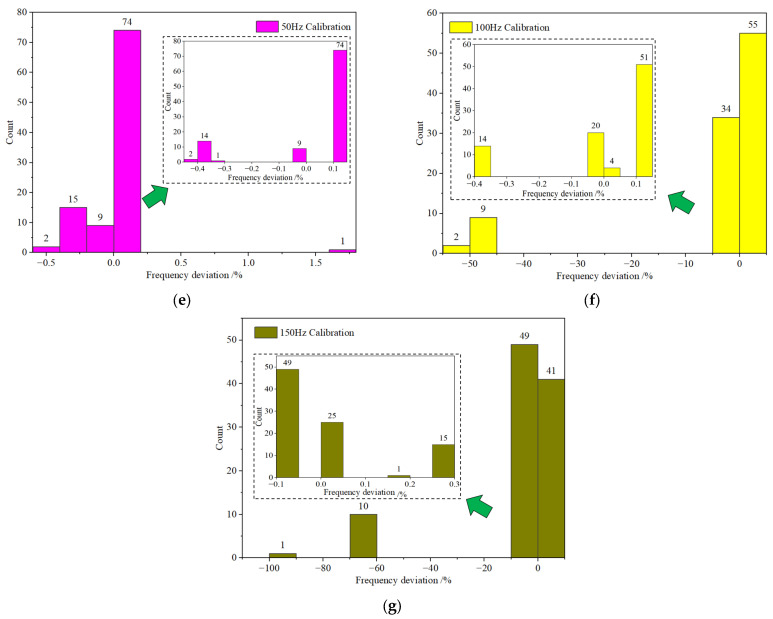
Statistical distribution of tested sensor frequency deviation at various test frequencies: (**a**) 2 Hz; (**b**) 5 Hz; (**c**) 10 Hz; (**d**) 20 Hz; (**e**) 50 Hz; (**f**) 100 Hz; (**g**) 150 Hz.

For a more comprehensive analysis of the frequency calibration outcomes, as illustrated in Figure 8, we meticulously tabulated the frequency deviations of the measured MS for each calibration frequency. Figure 8 demonstrates that the test results of certain MS exhibit significant deviations from the expected values at certain calibration frequencies. For instance, during the 5 Hz calibration, there are 11 MS with frequency deviations ranging between 800% and 1000%, considerably surpassing the established normal range. It was discerned that these 11 MS did not function correctly due to substantial biases present at each calibration frequency. Upon eliminating the aberrant values, the frequency deviation of the majority of MS at each calibration frequency fell within a narrow spectrum. For instance, at 10 Hz, the frequency deviation of 75 MS ranged from 0% to 0.2%. This pattern persisted across other calibration frequencies, underscoring the method’s efficacy in discerning non-operational MS. 

### 3.3. Amplitude Calibration Result Analysis

#### 3.3.1. Amplitude Deviation Analysis

**Figure 9:**
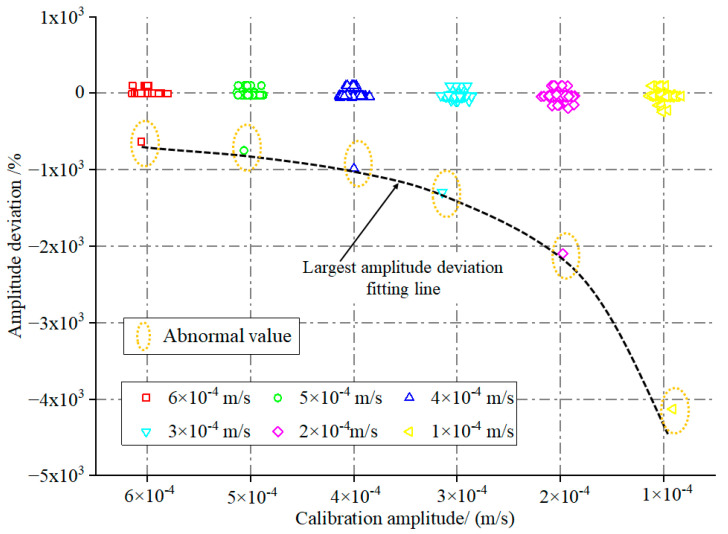
Statistical distribution of tested sensor amplitude deviation.

In total, 102 MS underwent testing on the CS18VLF, following the procedure outlined in Section 2.3.2. Subsequently, the amplitude deviation index was computed using Equation (2). As depicted in Figure 9, we conducted amplitude testing on 102 MS and computed their amplitude deviations using Equation (2). Analogously to the frequency calibration, we observed that the maximum amplitude deviation value escalates as the calibration amplitude decreases. Notably, at the 1 × 10^−4^ m/s calibration, the maximum amplitude deviation of one MS reaches −4000%. This trend could stem from the reduction in the amplitude during calibration, leading to a decrease in the denominator of Equation (2), consequently resulting in an elevation of the amplitude deviation. 

**Figure 10:**
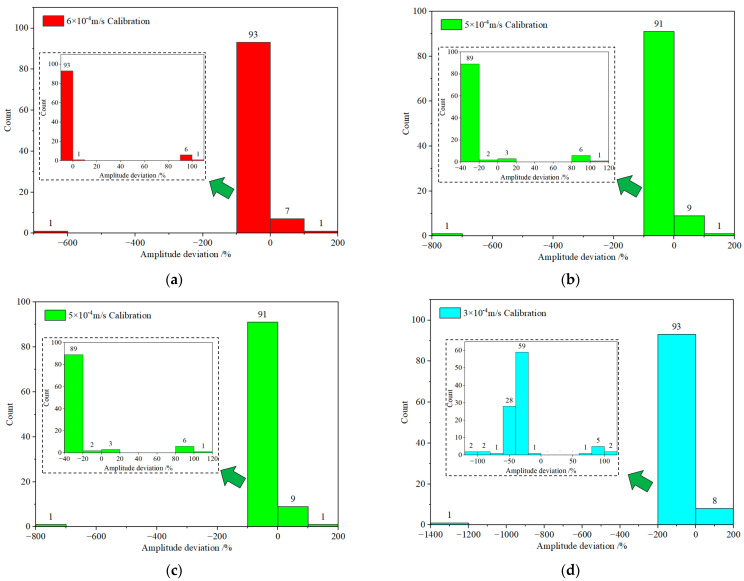

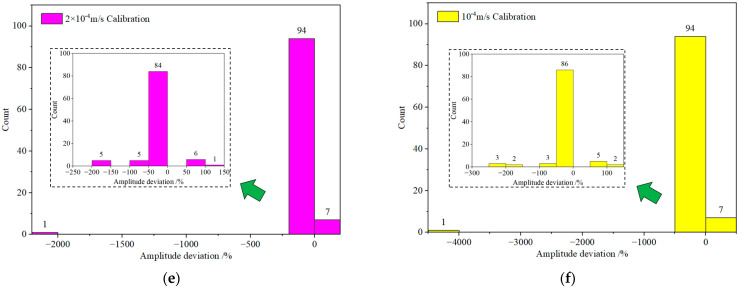
Statistical distribution of tested sensor amplitude deviation at various test amplitudes: (**a**) 6 × 10^−4^ m/s; (**b**) 5 × 10^−4^ m/s; (**c**) 4 × 10^−4^ m/s; (**d**) 3 × 10^−4^ m/s; (**e**) 2 × 10^−4^ m/s; (**f**) 1 × 10^−4^ m/s.

We graphed the outcomes separately for each calibration amplitude for a more granular analysis of the amplitude test results, as illustrated in Figure 10. This figure reveals that regardless of the calibration amplitude, the overwhelming majority of MS exhibit amplitude deviation values proximate to the norm, with only a small minority of MS demonstrating significant deviations from the expected range. Figure 10 illustrates the following observations for different calibration amplitudes: At the calibration amplitude of 6 × 10^−4^ m/s, 93 MS exhibit amplitude deviations ranging from −10% to 0; during the 5 × 10^−4^ m/s calibration, 89 MS display amplitude deviations between −40% and −20%; t the 4 × 10^−4^ m/s calibration, 62 MS manifest amplitude deviations within the range of −40% to −20%; with the calibration amplitude set at 3 × 10^−4^ m/s, 59 MS demonstrate amplitude deviations spanning from −40% to −20%; when calibrating at 2 × 10^−4^ m/s, 84 MS showcase amplitude deviations in the range of −50% to 0. Similarly, at the calibration amplitude of 1 × 10^−4^ m/s, 86 MS exhibit amplitude deviations between −50% and 0. These results imply that as the calibration amplitude diminishes, the number of MS that satisfy the given condition gradually decreases. Moreover, each calibration amplitude has a limited number of MS with amplitude deviations significantly beyond the normal range, signifying improper functionality. It is evident that the test can differentiate MS with abnormal measurement amplitudes. 

#### 3.3.2. Amplitude Linearity Analysis

**Figure 11:**
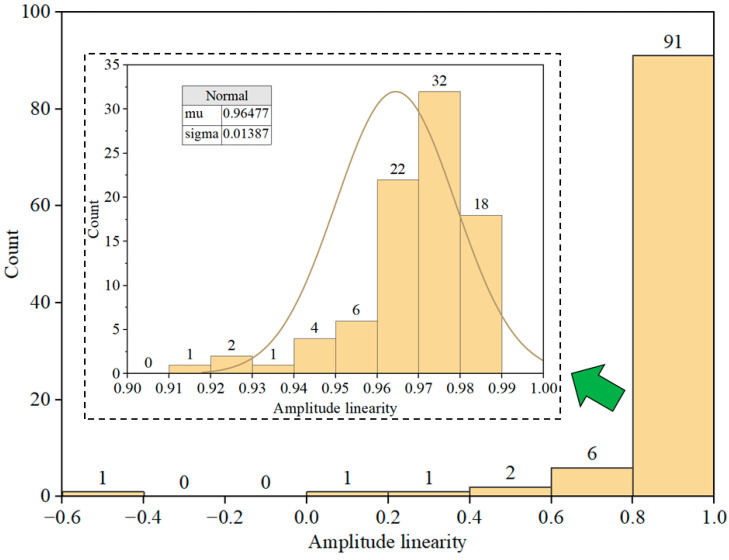
Statistical distribution of MS amplitude linearity.

Amplitude deviation analysis enables the assessment of accuracy in measuring different amplitudes using MS, while amplitude linearity analysis evaluates the consistency of measurements across varying amplitudes. The data source for amplitude linearity is identical to that of amplitude deviation. The amplitude linearity is calculated as demonstrated in Equation (3). As depicted in Figure 11, the statistical distribution results of amplitude linearity for the 102 MS are presented. Figure 11 shows that 91 out of the 102 MS exhibit amplitude linearity within the range of 0.8 to 1. Upon deeper analysis, it was observed that among these, 72 MS exhibit amplitude linearity between 0.96 and 0.99. This suggests that the majority of the MS demonstrate a strong level of amplitude linearity, affirming the stability and reliability of the amplitude testing for these MS.

## 4. Discussions

### 4.1. Determining the Discriminant Critical Value

The previous elucidates the equipment and procedure employed for calibrating MS. However, it is of paramount importance to establish criteria that facilitate the determination of the normal operational status of old MS. Twenty new MS were assessed using CS18VLF, and their amplitude–frequency calibration indices were computed employing Equations (1)–(3). Subsequently, the evaluative critical value was determined by comparing the discrepancies between the calibration indices of the new and old MS. The calibration method and process remain consistent with the previous description and will not be reiterated here. 

**Figure 12:**
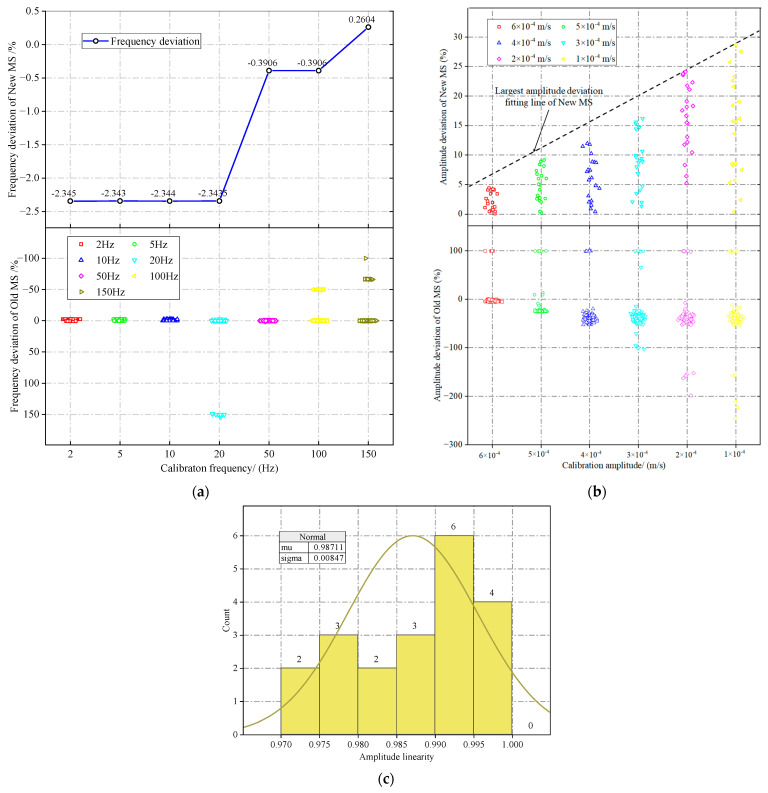
Comparative analysis of amplitude–frequency calibration results for new and old MS: (**a**) Comparative frequency deviation between new and old MS; (**b**) comparative amplitude deviation between new and old MS; (**c**) statistical distribution of amplitude linearity for new MS.

Figure 12a compares the frequency deviation calibration outcomes for old and new MS. The frequency deviation of the new MS falls within the range of 0.5% to −2.5%. Furthermore, for calibration frequencies of 2, 5, 10, and 20 Hz, their frequency deviations are −2.34%. With the escalation of the calibration frequency, the frequency deviation diminishes to −0.39% at 50 and 100 Hz and becomes 0.26% at 150 Hz. The old MS frequency deviation in Figure 12a eliminated the outliers marked in Figure 7. The frequency deviation of most old MS fluctuates between −5% and 5%. At 20 Hz calibration, the frequency deviation of a small number of old MS reached 150%; at 100 Hz calibration, it went −50%; and at 150 Hz, calibration reached −100%. Drawing upon the data analysis presented above, we conclude that the frequency measurement of the old MS is considered normal when the absolute value of the frequency deviation remains below 5%. Upon evaluating the old MS against this benchmark, it was found that 83 of them met the specified requirements. 

Figure 12b compares the amplitude deviations of old and new MS. Upon examining the amplitude calibration results of the new MS, it is evident that the largest amplitude deviation for each calibration amplitude exhibits a noticeable linearity. Specifically, this deviation escalates from 5% at 6 × 10^−4^ m/s to 30% at 1 × 10^−4^ m/s. This pattern can be attributed to the reduction in the denominator in Equation (2). Similarly, the amplitude deviation of the old MS has been adjusted by excluding the outliers identified in Figure 9. The amplitude deviation of the old MS exhibited a range from −250% to 100%. The positive deviation of their amplitude reached 100%, whereas the negative deviation increased with a decrease in the calibration amplitude, rising from −5% at 6 × 10^−4^ m/s to −250% at 1 × 10^−4^ m/s. Subsequent data analysis revealed that the majority of the old MS had an absolute amplitude deviation of less than 55%. Therefore, the established criterion for determining the normality of MS amplitude measurement is an absolute amplitude deviation below 55%. Upon evaluating the old MS against this criterion, it was found that 96 of them fulfilled the specified requirements. 

Figure 12c provides a visual representation of the statistical distribution of the results derived from the amplitude linearity calculation for the new MS. Notably, the amplitude linearity of all the new MS falls within the range of 0.97 to 1, indicating a robust correlation. By comparing this distribution with the information depicted in Figure 11, the critical value for amplitude linearity is determined to be 0.95. Consequently, based on this threshold, 78 of the old MS met the criteria of amplitude linearity.

### 4.2. Effect of Amplitude Deviation on Microseismic Localization

Microseismic source localization is a fundamental task within MMS. Various methods have been proposed, including the P-wave initial arrival method, the P-wave and S-wave time difference method, and the azimuth angle method (for three-component MS), to accurately determine the microseismic source position [28,29,30,31]. This paper employs the P-wave initial arrival method to demonstrate the impact of amplitude deviation on localization outcomes. The underlying principle of this method is to identify the point on the seismogram where a notable deviation from the background noise is observed, signifying the initial arrival of the P-wave [32]. After establishing the initial arrival time of the P-wave, the coordinates of the microseismic source are determined by combining the coordinates of the MS with the velocity of the P-wave. All microseismic source localization methods adhere to the fundamental principles of distance–time constraints, which can be expressed as follows:

(4) $$t-t_{0}=\int \frac{1}{v(s)}ds$$

where t is the initial arrival time of the P wave, $t_{0}$ is the time when the microseismic event happens, s is the wave propagation path from the source to the sensor, and v(s) is the wave velocity on the propagation path. As highlighted in Formula (4), the accurate determination of the initial arrival time of the P-wave holds paramount significance in achieving accurate microseismic source localization results. 

**Figure 13:**
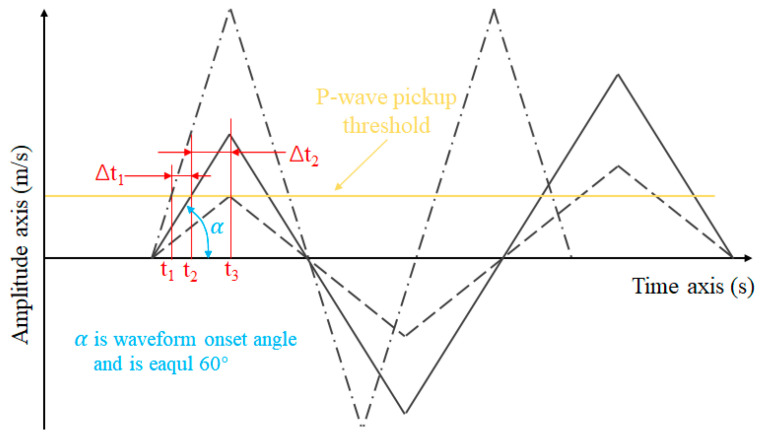
The P-wave initial arrival error due to amplitude deviation (The black dashed and dotted lines represent waveforms with amplitude deviations of 100% and −50%, respectively).

Given the complexity of actual vibration waveforms, a simplification was made using a triangular waveform (as shown in Figure 13) for computational analysis. As illustrated in Figure 13, in cases of positive amplitude deviation, the P-wave’s initial arrival time shifts to an earlier point, denoted as $t_{1}$. Conversely, when experiencing negative amplitude deviation, the P-wave’s initial arrival time shifts to a later point, represented by $t_{3}$. Notably, the extent of this shift in the P-wave’s arrival time corresponds directly to the absolute value of the amplitude deviation. In simpler terms, when there is a larger amplitude deviation, the P-wave’s arrival time shift becomes more noticeable compared to its expected timing. This emphasizes how amplitude deviation can significantly affect the accuracy of determining when the P-wave initially arrives, which impacts how precisely we can locate the source of the microseismic event. If the amplitude deviation becomes substantial, it could even make identifying the P-wave’s initial arrival difficult, potentially leading to the event going undetected.

To delve deeper into the impact of amplitude deviation on localization outcomes, a series of simplifications was involved to elucidate the relationship between amplitude deviation and the accuracy of microseismic source location. We considered the most ideal scenario for our discussion, where we have a total of four MS dedicated to positioning. At the same time, only one of these MS exhibits an amplitude error, while the remaining MS are considered error-free. As illustrated in Figure 13, using trigonometric relationships and referencing Equation (4), we infer the intricate relationship linking positioning errors, amplitude deviation, P-wave velocity, and the standard amplitude. The formulation describes these variables as follows:

(5) $$P_{e}=\frac{V_{p}\times A_{d}\times \delta_{a}}{\tan\alpha }$$

where $P_{e}$ is positioning error, m; $V_{p}$ is the p-wave velocity, m/s; $A_{d}$ is standard amplitude, m/s; $\delta_{a}$ is amplitude deviation; α is wave onset angle. 

**Figure 14:**
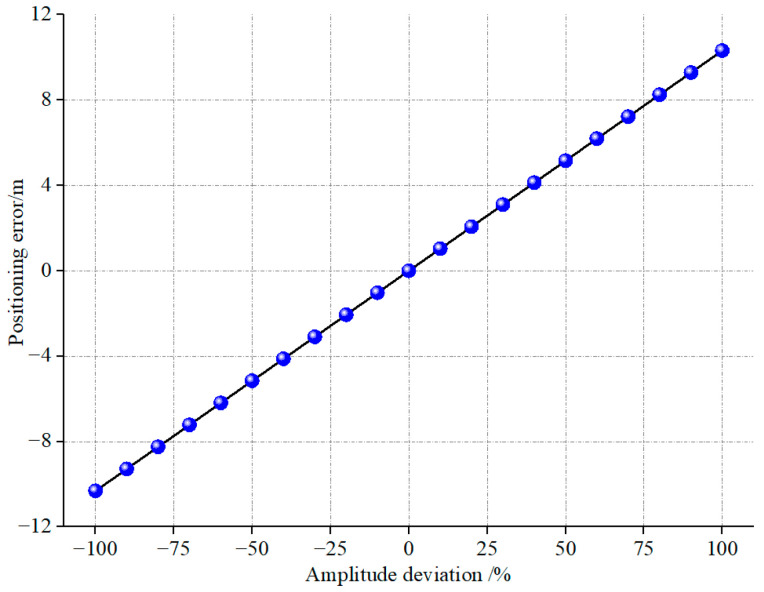
Positioning error caused by amplitude deviation.

Equation (5) uses assumed values for α, $V_{p}$, and $A_{d}$ (α = 60°, $V_{p}$ = 5500 m/s, $A_{d}$ = 6 × 10^−4^ m/s) to establish a mathematical relationship. This relationship elucidates the influence of the parameter $\delta_{a}$ on the quantity $P_{e}$, and its result is then graphically depicted in Figure 14. The figure portrays that within the context of the assumptions above when the amplitude deviation reaches 100%, the resulting positioning error extends to 10.3 m. It is important to note that this outcome is an oversimplified representation. The actual scenario is significantly more intricate, encompassing various factors contributing to the positioning error. 

### 4.3. Effect of Amplitude–Frequency Deviation on Microseismic Energy

Various methods exist for calculating the energy of a microseismic event, including the Gutenberg–Richter, energy density, duration of vibration, diagram integral, and discrete methods [33]. In this study, the Gutenberg–Richter method was selected to illustrate the impact of amplitude–frequency deviation on microseismic energy. The chosen method is rooted in wave propagation theory in an elastic medium. The vibration energy, E, can be expressed using the following equation:

(6) $$E=2\pi^{3}\rho \nu kr^{2}e^{2\gamma_{i}r}\sum_{i,k=1}^{n}{\left(A_{ik}f_{ik}\right)}^{2}\tau_{ik}$$

where ν is the propagation speed of the vibration wave, $A_{ik}$ and $f_{ik}$ is the amplitude and frequency of the wave, τ is the duration of the wave, k is the type of wave, P wave, and S wave, ρ is the density of the propagation cutoff, γ is damping coefficient of vibration wave, and r is the source of distance. 

In alignment with our prior discussions, we have chosen to simplify when analyzing the impact of amplitude bias on energy calculation results. Specifically, we assume a localization scenario that utilizes one sensor with amplitude error alongside three sensors that are assumed to be error-free. At the same time, the energy we discuss is the energy calculated by the sensor with amplitude error. A positive amplitude deviation results in an advance in the initial arrival time of the P wave, consequently advancing the onset time of the MS source. In such cases, this corresponds to a reduction in r in Equation (6). Conversely, when a negative amplitude deviation occurs, it causes a delay in the initial arrival time of the P wave, leading to a delayed calculation of the source onset time, and the r in Equation (6) becomes larger. Equation (6) shows that both the squared of r and the squared of $A_{ik}$ are positively correlated with E when other parameters remain unchanged. As a result, the energy calculation deviation induced by amplitude deviation is intricate, and under ideal circumstances, this energy deviation might even be negated or canceled out. 

Currently, numerous researchers have conducted extensive research in data mining, microseismic signal classification, and related areas based on microseismic monitoring databases [34]. However, it is essential to highlight that the development of methods to ensure the reliability and precision of microseismic sensors and systems remains an important and worthwhile research direction. This aspect is fundamental to the integrity of microseismic signal waveforms and the overall reliability of microseismic databases, serving as the cornerstone for subsequent in-depth analyses and discoveries. 

## 5. Conclusions

This study effectively addresses the research gap in the calibration of microseismic sensors and emphasizes the crucial importance of this calibration. The research systematically investigates and examines the microseismic monitoring system used in Chinese coal mines, thoroughly explaining the principles governing the monitoring and calibration of MS. The CS18VLF was constructed to promote research in this area, enabling a range of meticulous calibration experiments for MS. The key findings from this research can be summarized as follows:

(1) The comparison test between old and new MS using CS18VLF reveals that MS can experience failure after prolonged use in underground conditions. This failure is primarily characterized by significant deviations from the normal range in the measured frequency and amplitude. The methodology employed in this study has demonstrated its efficacy in identifying these faulty sensors.
(2) Using a comparative analysis of frequency deviation, amplitude deviation, and amplitude linearity between the old and new MS, the critical threshold for evaluating the effectiveness of MS was established. A sensor is deemed functional when its absolute frequency deviation is below 5%, absolute amplitude deviation is below 55%, and amplitude linearity exceeds 0.95.
(3) Under normal operating, the frequency deviation of the MS is significantly smaller than the amplitude deviation. Consequently, the effect of frequency deviation on microseismic energy can be deemed negligible.
(4) Simplified waveform analysis reveals a linear relationship between amplitude deviation and microseismic localization results. However, microseismic energy is influenced by both amplitude deviation and localization results. Therefore, pinpointing the precise impact of amplitude deviations on microseismic energy is challenging.

## Figures and Tables

**Table 1:** Performance parameters of APS113-AB.

Parameter	Performance
The driving force (sine peak)	133 N
Maximum displacement	158 mm
Maximum acceleration	13 m/s^2^
Frequency	DC~200 Hz
Maximum load	1.5 kg

## Data Availability

Not applicable.
